# Numeric Rating Scales Show Prolonged Post-exertional Symptoms After Orthostatic Testing of Adults With Myalgic Encephalomyelitis/Chronic Fatigue Syndrome

**DOI:** 10.3389/fmed.2020.602894

**Published:** 2021-01-27

**Authors:** C (Linda) M. C. van Campen, Peter C. Rowe, Freek W. A. Verheugt, Frans C. Visser

**Affiliations:** ^1^Stichting CardioZorg, Hoofddorp, Netherlands; ^2^Department of Pediatrics, Johns Hopkins University School of Medicine, Baltimore, MD, United States; ^3^Onze Lieve Vrouwe Gasthuis (OLVG), Amsterdam, Netherlands

**Keywords:** numeric rating scale, pain, fatigue, concentration, post-exertional malaise, fibromyalgia, orthostatic intolerance, myalgic encephalomyelitis/chronic fatigue syndrome

## Abstract

**Introduction:** Muscle pain, fatigue, and concentration problems are common among individuals with myalgic encephalomyelitis/chronic fatigue syndrome (ME/CFS). These symptoms are commonly increased as part of the phenomenon of postexertional malaise (PEM). An increase in the severity of these symptoms is described following physical or mental exercise in ME/CFS patients. Another important symptom of ME/CFS is orthostatic intolerance, which can be detected by head-up tilt testing (HUT). The effect of HUT on PEM has not been studied extensively. For this purpose, we assessed numeric rating scales (NRS) for pain, fatigue, and concentration pre- and post-HUT. As pain is a core symptom in fibromyalgia (FM), we subgrouped ME/CFS patients by the presence or absence of FM.

**Methods and Results:** In eligible ME/CFS patients who underwent HUT, NRS of pain, fatigue, and concentration were obtained pre-HUT, immediately after HUT, at 24 and 48 h, and at 7 days posttest. We studied 174 ME/CFS patients with FM, 104 without FM, and 30 healthy controls (HC). Values for all symptoms were unchanged for HC pre- and post-HUT. Compared with pre-HUT, the three NRS post-HUT were significantly elevated in both ME/CFS patient groups even after 7 days. NRS pain was significantly higher at all time points measured in the ME/CFS patients with FM compared with those without FM. In ME/CFS patients, the maximum fatigue and concentration scores occurred directly post-HUT, whereas pain perception reached the maximum 24 h post-HUT.

**Conclusion:** NRS scores of pain, fatigue, and concentration were significantly increased even at 7 days post-HUT compared with pre-HUT in ME/CFS patients with and without FM, suggesting that orthostatic stress is an important determinant of PEM.

## Introduction

Postexertional malaise (PEM) is one of the criteria of chronic fatigue syndrome (CFS) (1) and the cardinal feature of current case definitions for myalgic encephalomyelitis (ME)/CFS (2) and ME (3). PEM can include exacerbation of some or all of an individual's ME/CFS symptoms following physical or cognitive exertion or longitudinal neural strain (4) and leads to a decline in functional ability (5). As described by patients and supported by research, PEM is more than fatigue following a stressor (2) and can be described by patients as a postexertional crash, exhaustion, flare-up, collapse, debility, or setback. Studies have shown that PEM can involve flu-like feelings (6), the initiation or exacerbation of headache, muscle, or joint pain (6–8); increased cognitive dysfunction (short memory problems, prolonged time to process information) (6, 9, 10); increased gastrointestinal symptoms; orthostatic intolerance (OI) symptoms, such as lightheadedness/vertigo, sensory sensitivity (to light, sound, etc.) (6); sleep disturbances; and feelings of depression or anxiety (6, 7, 11).

Fibromyalgia (FM), a disease characterized by fatigue and prominent widespread musculoskeletal pain (12, 13), is highly prevalent in ME/CFS. A population-based study revealed that 94% of ME/CFS patients report muscle pain, and 84% report joint pain (14). In fact, there is a great overlap in symptoms between ME/CFS and FM.

Impairments in cognitive functioning, such as memory problems or concentration issues, are among the most frequently reported symptoms of ME/CFS. Patients classify the cognitive problems as equally debilitating compared with the physical symptoms that accompany this disease. One of the best-studied aspects of ME/CFS is cognition. In a meta-analysis of 50 studies using a total of 80 cognitive tests with 79 different scores, of 8 cognitive domains described, reaction time and attention were the only two domains with a moderate-to-large significant difference between ME/CFS patients and healthy controls (HC) (15). Thus, clinical observations suggest that concentration issues are an important part of the disease.

In a previous study in ME/CFS patients, we demonstrated that the orthostatic stress of a head-up tilt test (HUT) resulted in a significant reduction in cerebral blood flow (CBF) and that the blood-flow reduction was associated with the onset of worsening of pain, fatigue, and concentration problems (16)—all characteristic features of PEM. Therefore, we hypothesized that pain, fatigue, and concentration problems, as components of the PEM response, would be increased during the days following HUT. For this purpose, numeric rating scales (NRS) of pain, fatigue, and concentration were completed by ME/CFS patients before and directly after HUT and after 24 h, 48 h, and 7 days post-HUT. We subgrouped those with ME/CFS by the presence or absence of FM and compared them with HC.

## Materials and Methods

### Eligible Participants

Individuals diagnosed with ME/CFS, who underwent HUT at the Stichting CardioZorg between November 2014 and April 2018 because of a clinical suspicion of OI and in whom a complete set of the NRS was available, were included in this study. This cardiology clinic specializes in diagnosing and treating adults with ME/CFS. ME/CFS was considered present if participants met both the criteria for CFS (1) and ME (3), taking the exclusion criteria into account. For this purpose, a detailed history was taken, in which the symptoms of the ME and CFS criteria were asked for. Moreover, the presence of a psychiatric history was looked for in the referral letter of the general physician and in the medical specialty reports that patients had in their possession. During the first visit, ME/CFS patients were classified as having FM or not, designated in the manuscript as FM+ and FM-, respectively. FM was considered present when the diagnosis had been confirmed by a rheumatologist or when patients fulfilled the criteria of FM based on the American College of Rheumatology (ACR) FM questionnaire (17). In the present study, FM was considered part of the symptomatology of ME/CFS with more extensive and severe muscle pains in the FM+ ME/CFS individuals. From the ACR FM questionnaire, the widespread pain index (WPI) and the symptom severity scale score (SS scale score) were noted. For comparison, 30 HC underwent the same HUT and NRS collection. These HC were recruited from three sources: (a) announcements on ME/CFS patient advocacy websites, (b) posters in the medical clinic's office building, and (c) healthy acquaintances of the ME/CFS participants. Before entering the study, they were asked whether they had a chronic illness and whether they used medication. No formal assessment of the physical activity status of the HC was obtained.

The study was carried out in accordance with the Declaration of Helsinki. The use of clinical data for descriptive studies (PT1450) and the use of HC (P1411) were approved by the ethics committee of the Slotervaart Hospital, the Netherlands. All patients and HC gave written informed consent.

### HUT and CBF Measurements

The study and measurements were performed as described previously (16, 18, 19). Additional information can be found in the Supplementary Material.

### NRS for Pain, Fatigue, and Concentration

Patients and HC were presented with a paper with an NRS for pain with the numbers placed vertically. Participants were instructed to rate their pain level ranging from 0 to 10 (20–22). On the NRS for pain paper, anchors were given for each symptom at 0 = no pain, 1 = very mild pain, 4 = moderate pain, 8 = very strong pain, and 10 = the worst imaginable pain possible. An NRS was designed for fatigue and concentration, using anchors in line with those suggested by Borg and Noble for pain (21, 22). On the NRS for fatigue paper, anchors were given for each symptom at 0 = no fatigue, 1 = very mild fatigue, 4 = moderate fatigue, 8 = very strong fatigue, and 10 = the worst imaginable fatigue possible. On the NRS for concentration paper, anchors were given for each symptom at 0 = no concentration issues, 1 = very mild concentration issues, 4 = moderate concentration issues, 8 = very strong concentration issues, and 10 = the worst imaginable concentration issues possible. Thus, the higher the score, the more severe the impact of that symptom on the individual. Participants were asked to complete the NRS before the start of HUT and directly after HUT. The NRS paper was sent by e-mail to participants 24 h, 48 h, and 7 days after HUT.

### Statistical Analysis

Data were analyzed using the statistical package of Graphpad Prism version 8.2.4 (Graphpad software, La Jolla, California, USA). All continuous data were tested for normal distribution using the Kolmogorov–Smirnov normality test and presented as mean (standard deviation, SD) or as median (interquartile range, IQR) where appropriate. Variables that were not normally distributed were tested for skews with histograms. For variables with evenly distributed skews, we compared groups using an ordinary or mixed two-way ANOVA with *post hoc* Tukey correction where appropriate. Nominal data were compared using the chi-squared test with a 3 × 2 table. Paired data were analyzed using the paired *t*-test or Wilcoxon signed ranks test where appropriate. Groups were compared using the Kruskall Wallis test for unpaired data. A *post hoc* analysis with Dunn's test for multiple comparisons was done where significant differences were present. For non-parametric data within group comparisons, we used the Friedman test. Linear regression was performed to assess the relation between measurements (reduction in CBF vs. NRS scales of pain, fatigue, and concentration at all assessed points). A *p*-value of < 0.01 was considered significant.

## Results

A total of 400 patients visited the clinic during the study period. We excluded 32 individuals who did not meet criteria for the diagnosis of ME/CFS. Another 24 ME/CFS patients were excluded because they had no orthostatic intolerance in daily life (*n* = 8) or did not undergo a tilt test (*n* = 24). None of the patients had a psychiatric history with the exception of the psychiatric diagnosis of undifferentiated somatoform disorder. This diagnosis is often used by psychiatrists to characterize ME/CFS. A total of 336 individuals with diagnosed ME/CFS and OI underwent HUT and NRS assessments during the study period. Included patients did not participate in a study on working memory. Included patients were part of a previously reported study on pain pressure thresholds (164 in the fibromyalgia group and 84 in the group with no fibromyalgia). We excluded patients who did not complete the 24-h, 48-h, and 7-day NRS scores (*n*= 58; 17%). No patients used heart rate and/or blood pressure targeting drugs, and no patient had a body mass index (BMI) >37 kg/m^2^ and needed to be excluded for that reason. This left 278 ME/CFS patients to be analyzed. FM was present in 174 (63%) patients; 104 (37%) did not meet the criteria for FM. As part of the ME/CFS criteria, patients were asked about the presence of muscle pain complaints. In the FM- group of ME/CFS patients, 75/104 (72%) complained of muscle pains; in the FM+ group of ME/CFS patients, all patients 174/174 (100%) reported muscle pains. One hundred six (38%) ME/CFS patients used neuropathic pain medication with 20 (19%) in FM- patients and 86 (49%) in FM+ patients. No differences in scales were found in both groups with and without FM for patients using neuropathic pain medication and those who did not (data not shown). As expected, the WPI was significantly higher in FM+ ME/CFS patients compared with ME/CFS FM- patients [9 (6–12) vs. 4 (2–5), *p* < 0.0001]. The SS scale score was not significantly different between FM+ and FM- groups [8 (7–8) vs. 8 (7–9), *p* = 0.48]. In the FM- group (*n* = 104), 49 patients had a normal heart rate and blood pressure response to HUT, 24 had delayed orthostatic hypotension, and 31 had postural orthostatic tachycardia syndrome (POTS). No patients developed presyncope or syncope during HUT. In the FM+ group (*n*=174), 77 had a normal heart rate and blood pressure response to HUT, 24 had delayed orthostatic hypotension, and 73 had POTS. No patients developed presyncope or syncope during HUT. A chi-squared test on the division of hemodynamic HUT results vs. the absence or presence of FM (in a 3 × 2 table) was not statistically significant. No significant relations were found between the reduction in percentage of CBF and NRS for pain, fatigue, or concentration at all time points assessed (data not shown).

None of the HC had a chronic illness, and none used medication apart from the occasionally use of NSAIDs for pain. No pain medication was used on the day of the investigation.

Table 1 shows the demographic characteristics of the study population. ME/CFS patients with and without FM had higher supine heart rates compared to HC (both *p* < 0.0001) and higher end of HUT heart rates compared to HC (both *p* < 0.0001). ME/CFS patients without FM were significantly taller compared to ME/CFS patients with FM (*p* < 0.001), but weight and BMI did not differ. The male/female ratio was lower in the patient group with FM compared to without FM (*p* < 0.005). Disease duration did not differ between the ME/CFS patients without FM and with FM (*p* = 0.52). The ME/CFS subgroups were similar on all other variables.

**Table 1:** Demographic data and hemodynamic HUT results of the study population^*^.

	**Group 1** **HC (*n* = 30)**	**Group 2** **ME/CFS FM- (*n* = 104)**	**Group 3** **ME/CFS FM+ (*n* = 174)**	**One-way ANOVA and *post hoc* Tukey's test**
Male/female	7/23	18/86	12/162	Chi-square (3 × 2 table) =0.003
Age (years)	44(14)	40 (12)	38 (11)	*F* (2, 306) = 4.31; *p* < 0.05
Disease duration (years)^*^		10 (4-15)	9 (5-15)	Mann-Whitney test = 0.52
Height (cm)	174 (8)	174 (9)	171 (7)	*F* (2, 306) = 5.07; *p* < 0.05
Weight (kg)	75 (15)	74 (16)	73 (17)	*F* (2, 306) = 0.17; *p* = 0.85
BMI (kg/m^2^)	24.8 (4.5)	24.4 (4.9)	25.1 (5.6)	*F* (2, 306) = 0.55; *p* = 0.58
Heart rate supine (bpm)	64 (13)	74 (11)	76 (12)	*F* (2, 306) = 12.59; *p* < 0.0001. *Post hoc* tests: 1 vs. 2 *p* = 0.0001; 1 vs. 3 *p* < 0.0001 and 2 vs. 3 *p* = 0.56
Heart rate EOS (bpm)	78 (14)	98 (17)	101 (20)	*F* (2, 306) = 20.32; *p* < 0.0001. *Post hoc* tests: 1 vs. 2 *p* < 0.0001; 1 vs. 3 *p* < 0.0001 and 2 vs. 3 *p* = 0.29
SBP supine (mmHg)	135 (15)	137 (15)	136 (15)	*F* (2, 306) = 0.42; *p* = 0.42
SBP EOS (mmHg)	126 (15)	123 (18)	125 (19)	*F* (2, 306) = 0.34; *p* = 0.71
DBP supine (mmHg)	78 (7)	79 (8)	79 (7)	*F* (2, 306) = 0.22; *p* = 0.81
DBP EOS (mmHg)	81 (8)	82 (11)	83 (10)	*F* (2, 306) = 0.51; *p* = 0.60
CBF supine (ml)	623 (82)	611 (112)	623 (110)	*F* (2, 306) = 0.44; *p* = 0.65
CBF EOS (ml)	585 (77)	452 (95)	451 (93)	*F* (2, 306) = 29.57; *p* < 0.0001. *Post hoc* tests: 1 vs. 2 *p* < 0.0001; 1 vs. 3 *p* < 0.0001 and 2 vs. 3 *p* = 1.0
%CBF reduction at EOS	−6.0 (3.7)	−25.8 (8.9)	−27.6 (7.0)	*F* (2, 306) = 111.3; *p* < 0.0001. *Post hoc* tests: 1 vs. 2 *p* < 0.0001; 1 vs. 3 *p* < 0.0001 and 2 vs. 3 *p* = 0.15

*DBP, diastolic blood pressure; EOS, end of study; FM, fibromyalgia; HC, healthy controls; SBP, systolic blood pressure*.

* *data with median and IQR*.

**Figure 1:**
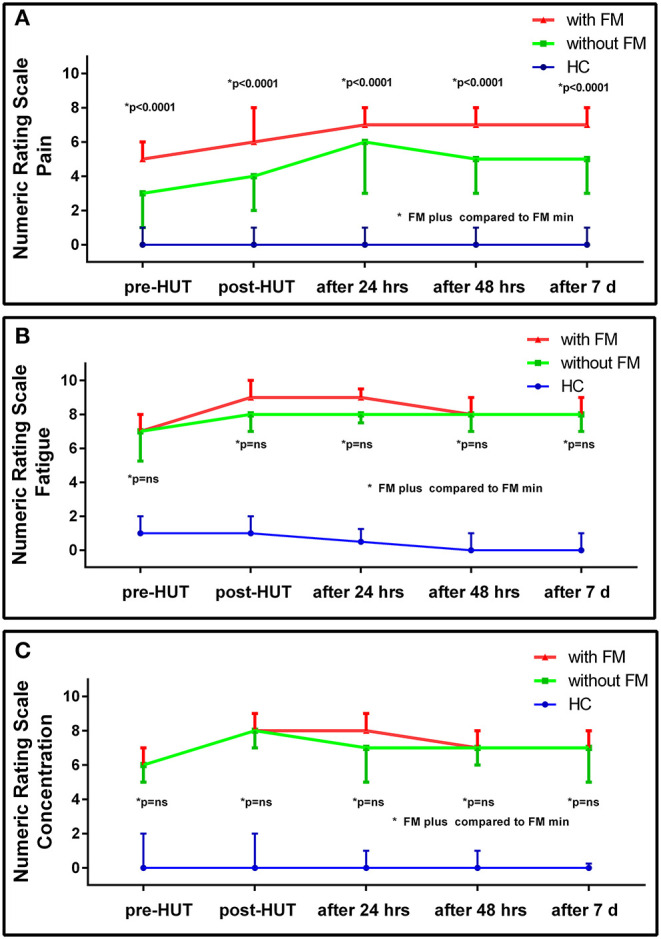
NRS for pain **(A)**, fatigue **(B)**, and concentration **(C)** pre- and post-HUT and after 24 h, 48 h, and 7 days in HC (*n* = 30), ME/CFS patients with FM (FM+; *n* = 174) and without FM (FM-; *n*=104).

Table 2 shows the NRS data pre- and post-HUT and after 24 h, 48 h, and 7 days for pain (Table 2A), fatigue (Table 2B), and concentration (Table 2C). NRS of pain, fatigue, and concentration for HC were all significantly lower than for ME/CFS patients (all *p* < 0.0001). At all measured time points (pre-HUT, post-HUT, after 24 h, after 48 h, and after 7 days), the NRS for pain differed significantly between the ME/CFS patients without FM and with FM (all *p* < 0.0001). Figure 1 shows the graphical presentation of the results of the NRS for pain **(A)**, for fatigue **(B)**, and for concentration **(C)** over time.

**Table 2A:** NRS scores by group for pain pre- and post-HUT and after 24 h, 48 h, and 7 days.

	**Group 1** **HC**	**Group 2** **ME/CFS FM-**	**Group 3** **with ME/CFS FM+**	**Kruskal–Wallis test with Dunn's multiple comparisons test**
NRS for pain pre-HUT	0 (0–1)	3 (1–5)	5 (3–6)	*X*^2^(2) = 68.75; *p* < 0.0001. *Post hoc* tests: 1 vs. 2 *p* < 0.0001; 1 vs. 3 *p* < 0.0001 and 2 vs. 3 *p* < 0.0001
NRS for pain post-HUT	0 (0–1)	4 (2–6)	6 (4–8)	*X*^2^(2) = 79.39; *p* < 0.0001. *Post hoc* tests: 1 vs. 2 *p* < 0.0001; 1 vs. 3 *p* < 0.0001 and 2 vs. 3 *p* < 0.0001
NRS for pain 24 h	0 (0–1)	6 (3–7)	7 (6–8)	*X*^2^(2) = 85.23; *p* < 0.0001. *Post hoc* tests: 1 vs. 2 *p* < 0.0001; 1 vs. 3 *p* < 0.0001 and 2 vs. 3 *p* < 0.0001
NRS for pain 48 h	0 (0–1)	5 (3–7)	7 (5.8–8)	*X*^2^(2) = 90.55; *p* < 0.0001. *Post hoc* tests: 1 vs. 2 *p* < 0.0001; 1 vs. 3 *p* < 0.0001 and 2 vs. 3 *p* < 0.0001
NRS for pain 7 days	0 (0–1)	5 (3–7)	7 (5–8)	*X*^2^(2) = 81.04; *p* < 0.0001. *Post hoc* tests: 1 vs. 2 *p* < 0.0001; 1 vs. 3 *p* < 0.0001 and 2 vs. 3 *p* < 0.0001

*FM, fibromyalgia; HC, healthy controls; HUT, head-up tilt test; NRS, numeric rating scale. With equal skews a mixed two-way ANOVA could be performed analyzing both patient groups (without FM and with FM) and all five in subject variables (pre-HUT, post-HUT, after 24 h, after 48 h and after 7 days) on pain: F (4, 1375)= 0.21; p = 0.93*.

**Table 2B:** NRS scores by group for fatigue pre- and post-HUT and after 24 h, 48 h, and 7 days.

	**Group 1** **HC**	**Group 2** **ME/CFS FM-**	**Group 3** **with ME/CFS FM+**	**Kruskal–Wallis test with Dunn's multiple comparisons test**
NRS for fatigue pre-HUT	1 (0–2)	7 (5.3–8)	7 (6–8)	*X*^2^(2) = 81.56; *p* < 0.0001. *Post hoc* tests: 1 vs. 2 *p* < 0.0001; 1 vs. 3 *p* < 0.0001 and 2 vs. 3 *p* = 0.20
NRS for fatigue post-HUT	1 (0–2)	8 (7–9)	9 (8–10)	*X*^2^(2) = 87.41; *p* < 0.0001. *Post hoc* tests: 1 vs. 2 *p* < 0.0001; 1 vs. 3 *p* < 0.0001 and 2 vs. 3 *p* = 0.10
NRS for fatigue 24 h	0.5 (0–1.3)	8 (8–9)	9 (8–9)	*X*^2^(2) = 74.27; *p* < 0.0001. *Post hoc* tests: 1 vs. 2 *p* < 0.0001; 1 vs. 3 *p* < 0.0001 and 2 vs. 3 *p* = 0.34
NRS for fatigue 48 h	0 (0–1)	8 (7–9)	8 (8–9)	*X*^2^(2) = 74.16; *p* < 0.0001. *Post hoc* tests: 1 vs. 2 *p* < 0.0001; 1 vs. 3 *p* < 0.0001 and 2 vs. 3 *p* = 0.31
NRS for fatigue 7 days	0 (0–1)	8 (7–8)	8 (7–9)	*X*^2^(2) = 73.97; *p* < 0.0001. *Post hoc* tests: 1 vs. 2 *p* < 0.0001; 1 vs. 3 *p* < 0.0001 and 2 vs. 3 *p* = 0.20

*FM, fibromyalgia; HC, healthy controls; HUT, head-up tilt test; NRS, numeric rating scale. With equal skews a mixed two-way ANOVA could be performed analyzing both patient groups (without FM and with FM) and all five in subject variables (pre-HUT, post-HUT, after 24 h, after 48 h, and after 7 days) on fatigue: F (4, 1375) = 0.47; p = 0.76*.

**Table 2C:** NRS scores by group for concentration pre- and post-HUT and after 24 h, 48 h, and 7 days.

	**Group 1** **HC**	**Group 2** **ME/CFS FM-**	**Group 3** **with ME/CFS FM+**	**Kruskal–Wallis test with Dunn's multiple comparisons test**
NRS for concentration pre-HUT	0 (0–2)	6 (5–7)	6 (5–7)	*X*^2^(2) = 76.70; *p* < 0.0001. *Post hoc* tests: 1 vs. 2 *p* < 0.0001; 1 vs. 3 *p* < 0.0001 and 2 vs. 3 *p* = 1.0
NRS for concentration post-HUT	0 (0–2)	8 (7–9)	8 (7–9)	*X*^2^(2) = 83.67; *p* < 0.0001. *Post hoc* tests: 1 vs. 2 *p* < 0.0001; 1 vs. 3 *p* < 0.0001 and 2 vs. 3 *p* = 0.24
NRS for concentration 24 h	0 (0–1)	7 (5–8)	8 (7–9)	*X*^2^(2) = 74.66; *p* < 0.0001. *Post hoc* tests: 1 vs. 2 *p* < 0.0001; 1 vs. 3 *p* < 0.0001 and 2 vs. 3 *p* = 0.06
NRS for concentration 48 h	0 (0–1)	7 (6–8)	7 (6–8)	*X*^2^(2) = 73.04; *p* < 0.0001. *Post hoc* tests: 1 vs. 2 *p* < 0.0001; 1 vs. 3 *p* < 0.0001 and 2 vs. 3 *p* = 0.22
NRS for concentration 7 days	0 (0–0.3)	6 (5–8)	7 (6–8)	*X*^2^(2) = 71.25; *p* < 0.0001. *Post hoc* tests: 1 vs. 2 *p* < 0.0001; 1 vs. 3 *p* < 0.0001 and 2 vs. 3 *p* = 0.44

*FM, fibromyalgia; HC, healthy controls; HUT, head-up tilt test; NRS, numeric rating scale. With equal skews a mixed two-way ANOVA could be performed analyzing both patient groups (without FM and with FM) and all five in subject variables (pre-HUT, post-HUT, after 24 h, after 48 h, and after 7 days) on concentration: F (4, 1375) = 0.26; p = 0.90*.

A subgroup analysis shows a difference in pre-HUT NRS for pain between ME/CFS patients with FM using neuropathic pain medication compared to those without. Despite the use of neuropathic analgesics, in the ME/CFS patient group with FM, the median NRS for pain was significantly higher in the patients using neuropathic analgesics than without neuropathic analgesics [median 6 (4–7) vs. 4 (2–6), *p* < 0.0001]. In the ME/CFS FM- group, there was no significant difference in the NRS for pain between the patients using neuropathic analgesics or not (*p* = 0.07). The NRS for fatigue did not differ significantly between the two ME/CFS groups at any time point (*p* ranging from 0.08 to 0.02), nor did the NRS for concentration differ at any time point (*p* ranging between 0.33 and 0.01). There was no difference in the NRS for fatigue or concentration in the ME/CFS patients using neuropathic analgesic medication or not (data not shown). There were no significant differences between males and females in the groups with and without FM for the NRS data on pain, fatigue, or concentration (data not shown).

**Figure 2:**
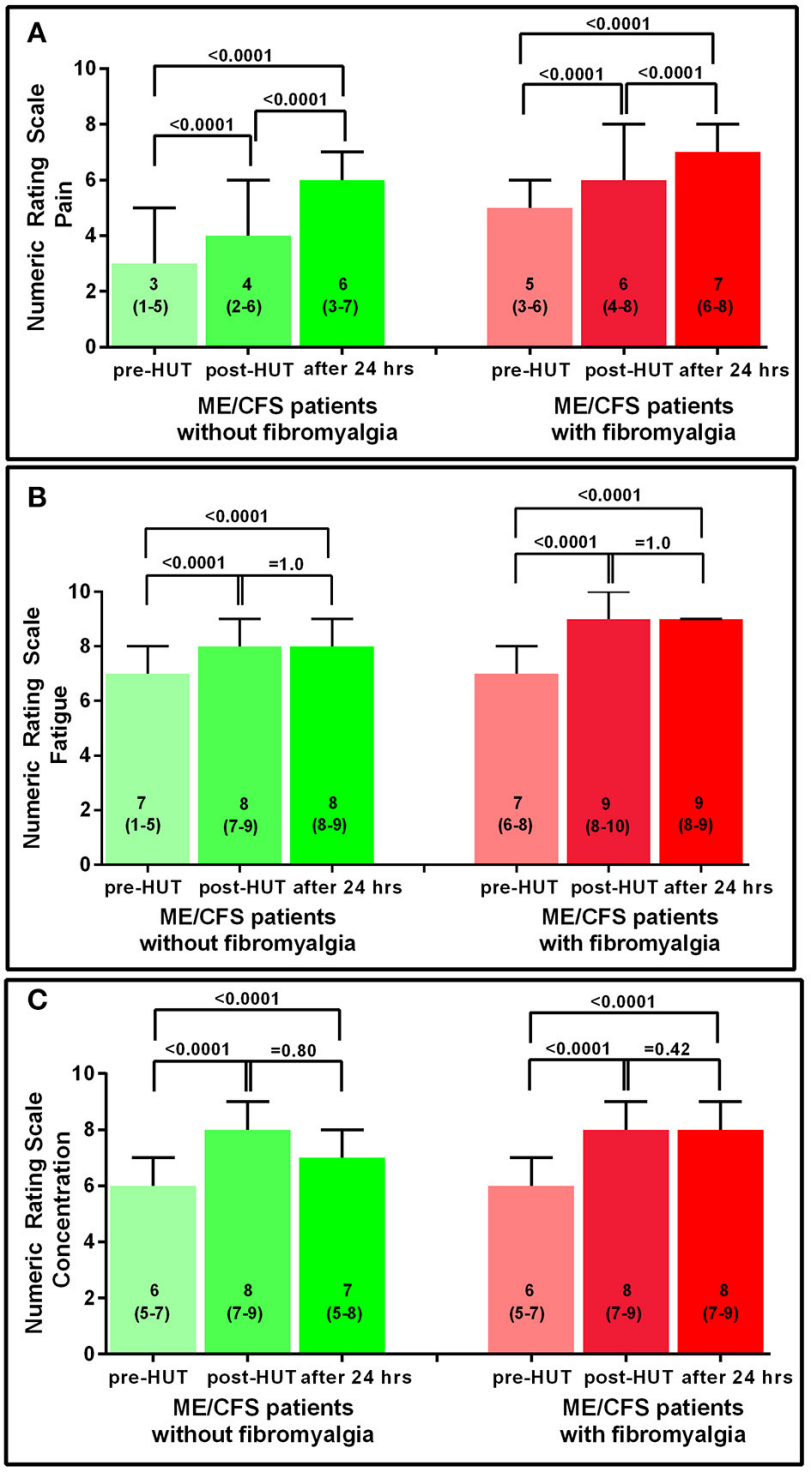
NRS for pain **(A)**, fatigue **(B)**, and concentration **(C)** pre-HUT, post-HUT, and after 24 h for ME/CFS patients with and without FM.

Figure 2 shows the results of the NRS for the two ME/CFS patient groups pre- and post-HUT and after 24 h for pain, fatigue, and concentration. In both patient groups, the maximal rating for pain was reached at 24 h with significant differences between pre- and post-HUT and between post-HUT and 24 h posttest (all *p* < 0.0001). Tilt testing was associated with a significant increase in fatigue and concentration problems in both ME/CFS patient groups, but we did not identify a progressive increase over the following 24 h.

**Figure 3:**
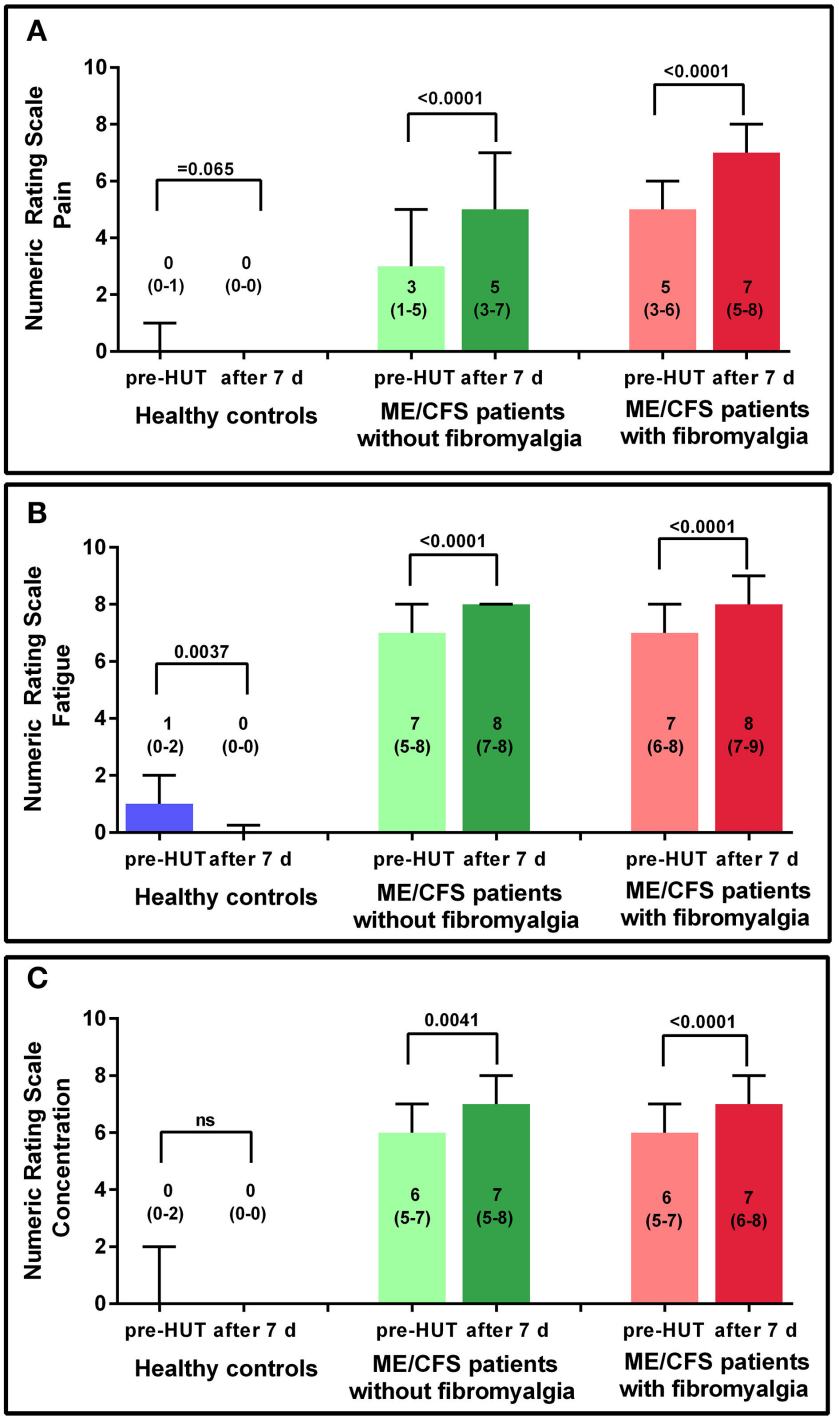
NRS for pain **(A)**, fatigue **(B)**, and concentration **(C)** pre-HUT and after 7 days for HC, and ME/CFS patients with and without FM.

Figure 3 shows the results of the NRS for HC and the 2 ME/CFS patient groups pre-HUT and at 7 days post-HUT for pain, fatigue, and concentration. In HC only, the NRS for fatigue decreased significantly (*p* = 0.004). In ME/CFS patients with and without FM, all three NRS scores were significantly higher 7 days post-HUT compared with pre-HUT (*p* ranging from 0.004 to < 0.0001).

**Figure 4:**
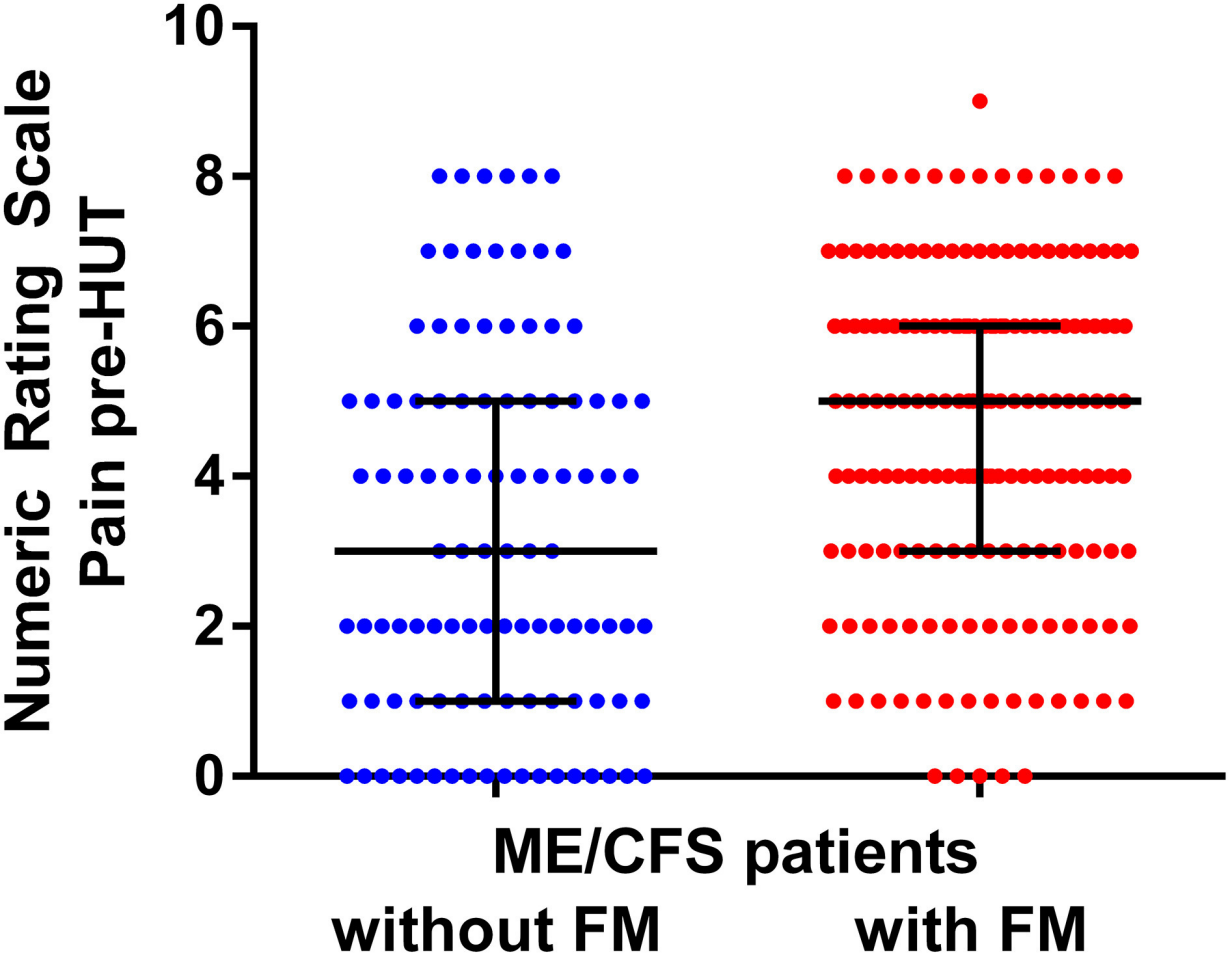
NRS for pre-HUT pain scores in ME/CFS patients with FM (*n* = 174) and without FM (*n* = 104).

Figure 4 shows the individual patient data points of the pre-HUT NRS of pain as given in Figure 2. Although there is a significant difference between the ME/CFS patients with and without FM, there is a large overlap of the pain NIRS scores.

CBF decline was 27 (8%) in the complete group. No significant relation was found between CBF reduction and differences in NRS for pain, fatigue, or concentration (data not shown).

## Discussion

To the best of our knowledge, this is the first study in ME/CFS patients to examine the influence of HUT on self-reported symptoms of PEM. We used NRS to assess changes in the severity of characteristic PEM symptoms of pain, fatigue, and concentration and to track the duration of these symptoms after the orthostatic stress imposed by a HUT. The study had several novel findings. First, scores for pain, fatigue, and concentration were significantly higher at baseline (pre-HUT) in all ME/CFS patients compared with HC. Second, all three NRS scores were significantly higher at all time points after HUT compared with pre-HUT in both ME/CFS patients groups. Third, ME/CFS patients with FM had significantly higher NRS pain scores than ME/CFS patients without FM at all time points. Fourth, maximum fatigue and concentration abnormalities occurred directly post-HUT, whereas pain perception reached a maximum 24 h posttest with an additional increase in pain scores from directly post-HUT to 24 h after testing. Fifth, PEM as defined by higher NRS scores of pain, fatigue, and concentration still persisted 7 days after HUT. Finally, no relation was found with the reduction in CBF during HUT and a difference in either of the NRS scales at all time points, and also, no relation was found with the maximal observed difference in NRS.

PEM has been described to occur after physical exercise (7, 23, 24), after cognitive efforts (25–27), and after emotional distress (28), and symptoms can persist for at least 24 h after a neuromuscular strain (4). Worsening of symptoms as part of PEM by upright posture seems to suggest that orthostatic stress is able to elicit PEM (2, 29) as was reported in a large patient survey in which 64.5% of included patients reported the presence of PEM with positional changes, and more than half of those most or all of the time (30). Although Ocon et al. describe deterioration of cognitive function during HUT in ME/CFS patients with orthostatic intolerance and POTS, this study did not address the persistence or increase of the cognitive dysfunction (PEM) in the hours, days, or weeks after the orthostatic stress test (10).

Blackwood et al. describe the onset of PEM occurring shortly after the trigger (31), and several studies describe a more prolonged interval between activity and aggravation of PEM (32–34). The survey by Chu et al. addresses PEM after physical and cognitive exertion and after emotional distress (28). They found that the onset of PEM symptomatology varied between an immediate onset and an onset of more than 24 h later. Importantly, a large number of patients reported a variable onset of PEM. The variable onset was confirmed by Holtzman et al., where immediate onset was reported in 72.3%, and delayed onset of PEM was reported in 91.4% (30). Half of the included patient group in this report had signs of PEM 1–2 days after the trigger, and even about 10% reported PEM more than 5 days after the eliciting trigger. In the present study, we observed that mean NRS scores on pain, fatigue, and concentration were immediately and significantly increased after the orthostatic stress compared with pretest values. Nevertheless, 111 (40%) patients showed increased NRS for pain 24 h posttest or later. Similarly, 58 (21%) patients showed no increase in the NRS for fatigue immediately after the HUT, and 69 (25%) showed no increase in the NRS for concentration directly after the HUT. This also indicates heterogeneity in the onset of PEM symptoms after the initial stressor, similar to other studies (28, 30).

HC report recovery within 1 or 2 days following physical or cognitive exertion. In contrast, < 31% of ME/CFS patients reported having returned to their prestressor baseline state after 1 to 2 days, and 60% of ME/CFS patients still experienced PEM symptoms after 1 week (2, 6, 7, 26). Our study shows that all three mean NRS scores were still significantly higher 1 week after the orthostatic stressor compared with their pretest scores. Therefore, our data suggest that orthostatic stress testing results in a prolonged duration of PEM symptomatology. So far, PEM has been an ill-defined symptom. Possibly, the findings of our study may result in a further quantification of the duration and severity of PEM. This needs to be studied in the future. Whether orthostatic stress testing differs from exercise stress testing in regard to onset, severity, and duration of PEM symptoms needs to be studied further.

In a recent study, we show that worsening of pain, increased fatigue, decreased concentration, and increased dizziness or light-headedness were all experienced significantly more frequently during HUT by those with ME/CFS compared to HC (16). The increased symptoms were associated with a statistically and clinically significant decrease in CBF compared to HC. Extending these findings, we show that working memory function, as assessed by the *n*-back test, decreased immediately after HUT (35). Furthermore, we show a decrease in pressure pain thresholds (PPT) immediately after orthostatic stress similar to PPT changes in ME/CFS patients after an exercise stressor (36). We, therefore, hypothesize that the symptom perception increase as demonstrated by the increases in the severity of NRS ratings of pain, fatigue, and concentration increase, is related to the observed reduction in CBF. We did not identify a relation between the degree of CBF reduction and changes in NRS scores despite a CBF reduction of at least 13% (16). It is possible that a relationship exists between CBF and symptom severity, but this may not be evident above a certain threshold of CBF reduction. Moreover, the scales used in this study might not be sensitive enough to measure the full range of changes in symptoms, thereby reducing the opportunity to see a correlation with CBF. The hypothesis that CBF influences symptom reporting could be tested by applying lower body compression with positive pressure during HUT to see if this reduces venous pooling and the degree of fall in CBF, and is then followed by a reduction in the intensity and duration of PEM after orthostatic stress.

In the present study, FM was considered part of the symptomatology of ME/CFS with more extensive and severe muscle pains as in FM negative ME/CFS individuals. Whether FM has the same underlying pathophysiology as ME/CFS has been discussed over many decades. Some authors have opinioned that FM and ME/CFS cannot be differentiated (37, 38). Wessely and Hotopf say, “We conclude by suggesting that fibromyalgia is one of many medically unexplained syndromes which have more similarities than differences between them” (p. 434). Other authors have identified differences between ME/CFS patients with and without the presence of FM with regard to levels of substance P (39, 40), cognitive abnormalities and dysfunction (41), plasma prolactin after stimulation (42), balance abnormalities with standing (43), and abnormal sleep dynamics (44). Moreover, a difference in the prevalence of a viral trigger was found between CFS/ME patients with and without the presence of FM (45), together with a difference in the severity of PEM (46). Applying the new systemic exertion intolerance disease (SEID) criteria, in which pain is not included as a cardinal symptom, Jason et al. find that SEID patients with FM were more severely disabled than patients without FM (47). These issues are discussed in more detail in a recent review by Natelson et al. (48). Summarizing, the abovementioned data suggest that there may be a different underlying pathophysiology, but further studies are needed to clarify the true nature of the differences. Indeed, Castro-Marrero et al. suggest the use of five different comorbidity clusters of ME/CFS patients (49), in which FM is included in the first cluster. Finally, Blitshteyn and Chopra suggest that it may be more logical, beneficial, and therapeutically effective to stratify FM and CFS/ME patients into more specific diagnoses in the group of the so called “chronic disorders associated with fatigue” (50). Although there may be differences in the pathophysiology of FM vs. ME/CFS, the clinical distinction is difficult. This is highlighted in Figure 4. Based on the NRS of pain, no reliable prediction can be made to discriminate between ME/CFS patients with and without FM due to the largely overlapping NRS values.

## Limitations

Follow-up NRS ratings may have been influenced by ME/CFS patients remembering previous ratings. On the other hand, we have previously shown in ME/CFS patients that memory, as assessed by *n*-back testing, is diminished post-HUT compared with pre-HUT. This may reduce carryover effects of NRS ratings. In this study, a numeric rating scale ranging from 0 to 10 was used. A large variety of rating scales and anchors are used in previous studies: For an overview the systematic literature, see the review of Hjermstad et al. (51). The conclusion of the authors is that an NRS of 11 points (0 to 10), is adequate. Nevertheless, in the present study, pre-HUT NRS values for fatigue were high (with a median of 7). This elevated baseline score likely limited the ability to detect a substantial change in fatigue symptoms post HUT, implying that this rating scale might be less sensitive to an increase in symptoms (due to a ceiling effect). A further study is needed to determine whether these high NRS scores are also applicable to the home situation or are related to the stress of the visit in our clinic. If they are also applicable to the home situation, other measures would be needed to capture true increases in symptom severity among those with high baseline NRS scores. We only studied ME/CFS patients undergoing HUT because of a clinical suspicion of OI. It is unknown whether outcomes would be different in ME/CFS patients without OI although this group without OI represents only a small minority of those with ME/CFS as 90% of our ME/CFS participants exceed the normal limits for CBF reduction during tilt. Our data on the influence of an orthostatic stressor on PEM need to be replicated by others, possibly using even longer follow-up periods in these ME/CFS patient groups to gain more information on PEM duration.

## Conclusions

NRS for pain, fatigue, and concentration were significantly increased up to 7 days after orthostatic stress testing in ME/CFS patients. NRS for pain in patients with FM were all significantly higher than in patients without FM. Our data show that an orthostatic stressor is an important determinant of PEM.

## Data Availability Statement

The raw data supporting the conclusions of this article will be made available by the authors, without undue reservation.

## Ethics Statement

The studies involving human participants were reviewed and approved by Ethics committee of the Slotervaart Hospital, the Netherlands. The patients/participants provided their written informed consent to participate in this study.

## Author Contributions

CC and FCV conceived the study and collected the data. CC performed the primary data analysis. PR, FWAV, and FCV performed secondary data analyses. All authors were involved in the drafting and review of the manuscript.

## Conflict of Interest

The authors declare that the research was conducted in the absence of any commercial or financial relationships that could be construed as a potential conflict of interest.
