# Supplementary material for: Numeric Rating Scales Show Prolonged Post-exertional Symptoms After Orthostatic Testing of Adults With Myalgic Encephalomyelitis/Chronic Fatigue Syndrome
Source: Front Med (Lausanne). 2021 Jan 27;7:602894. doi: 10.3389/fmed.2020.602894 (PMC7874746; doi:10.3389/fmed.2020.602894)
Supplement: Supplementary file 1 [file Data_Sheet_1.PDF]

Supplementary Material

1 Supplementary Data

1.1 Head-up tilt table test:

The HUT was performed as described previously (van Campen et al. 2018). Briefly, testing was conducted at least 3 hours after a light meal. Participants were encouraged to ingest an ample amount of fluid on the day of the procedure, but did not drink fluids in the 2 hours before the test. Participants were studied in a climate-controlled room where the temperatures ranged from 22-24^0 C. Individuals were studied in the supine position for 15 minutes, and for 30 minutes in the upright position (70-degrees). The test was ended after 30 minutes, at the request of the patient, or if the individual developed syncope or pre-syncope.

Heart rate (HR), systolic and diastolic blood pressures (SBP and DBP) were continuously recorded by finger plethysmography using the Nexfin device (BMeye, Amsterdam, NL) (Eeftinck Schattenkerk et al. 2009; Martina et al. 2012). An independent radio-controlled clock was used to mark the starting time of HR and BP recordings as well as the time of the start of tilting. HR and BP data were extracted from the Nexfin device and imported into an Excel spreadsheet. Supine HR and BP data were calculated from the last minute data before tilting. Upright HR and BP data were calculated from the last minute data of the upright position. HR and BP responses during the HUT were classified according to consensus guidelines, like orthostatic hypotension (a decrease of over 20 mmHg in systolic blood pressure and over 30 mmHg in case of a systolic blood pressure over 140 mmHg, or a decrease of 10 mmHg in diastolic blood pressure) and postural orthostatic tachycardia syndrome (a sustained increase of at least 30 bpm within 10 minutes, without a significant decrease in BP) (Fedorowski et al. 2009; Freeman et al. 2018; Freeman et al. 2011; Sheldon et al. 2015). Nasal prongs were placed to measure expired carbon dioxide (CO2) concentrations. For the tilt testing component, individuals being treated with medication that could alter HR or BP (beta-adrenergic antagonists, midodrine, fludrocortisone, desmopressin, pyridostigmine bromide, anti-hypertensive medications, or ivabradine) were excluded from this analysis. Individuals being treated with selective serotonin reuptake inhibitors or serotonin norepinephrine reuptake inhibitors continued to take these medications.

1.2 Cerebral blood flow

Internal carotid artery and vertebral artery Doppler flow velocity frames were acquired by one operator (FCV), using a Vivid-I system (GE Healthcare, Hoevelaken, the Netherlands) equipped with a 6–13 MHz linear transducer. Flow data of the internal carotid artery (ICA) on the right and on the left side were obtained ~1.0–1.5 cm distal to the carotid bifurcation and of the vertebral artery (VA) on the right and on the left side, data were obtained at the C3-C5 level. Care was taken to ensure the insonation angle was less than 60 degrees, that the sample volume was positioned in the center of the vessel and that it covered the width of the vessel. High resolution B mode images, color Doppler images and the Doppler velocity spectrum (pulsed wave mode) were recorded in one frame. The order of imaging was fixed: left internal carotid artery (ICA), left vertebral artery (VA), right internal carotid artery (ICA), and right vertebral artery (VA). At least two consecutive series of six frames per artery were recorded. The recording time intervals of the first and last imaged artery were noted and

these times were corrected to the times of a radio clock, setting the start of tilt at 0 min. Heart rate and blood pressures of the echo recording time intervals were averaged. In the supine position, image acquisition started 8 (2) min prior to tilting (supine data) and during the upright position acquisition started at 10 (4) min. Based on data from healthy controls during a 30-minute 70 degree head-up tilt, we defined an abnormal reduction in CBF as a > 13% decline during the tilt compared to the supine values (van Campen et al. 2020).

Blood flows of the internal carotid and vertebral arteries were calculated offline by an investigator (CMCvC) who was unaware of the patient severity status and unaware of the hemodynamic outcome of the head-up tilt test. Vessel diameters were manually traced by CMCvC on B-mode images, from the intima to the opposite intima. Surface area was calculated: the peak systolic and end diastolic diameters were measured, and the mean diameter was calculated as: mean diameter=(peak systolic diameter×1/3) + (end diastolic diameter×2/3) (Sato et al. 2011). Blood flow in each vessel was calculated from the mean blood flow velocities times the vessel surface area and expressed in ml/min. Flow in the individual arteries was calculated in 3-6 cardiac cycles and data were averaged. Total cerebral blood flow was calculated by adding the flow of the four arteries. We previously demonstrated that this methodology had good intra- and inter-observer variability (van Campen et al. 2018).

References supplementary data

Eeftinck Schattenkerk DW et al. (2009) Nexfin noninvasive continuous blood pressure validated against Riva-Rocci/Korotkoff Am J Hypertens 22:378-383 doi:10.1038/ajh.2008.368

Fedorowski A, Burri P, Melander O (2009) Orthostatic hypotension in genetically related hypertensive and normotensive individuals J Hypertens 27:976-982 doi:10.1097/hjh.0b013e3283279860

Freeman R, Abuzinadah AR, Gibbons C, Jones P, Miglis MG, Sinn DI (2018) Orthostatic Hypotension: JACC State-of-the-Art Review J Am Coll Cardiol 72:1294-1309 doi:10.1016/j.jacc.2018.05.079

Freeman R et al. (2011) Consensus statement on the definition of orthostatic hypotension, neurally mediated syncope and the postural tachycardia syndrome AutonNeurosci 161:46-48 doi:S1566-0702(11)00035-X [pii];10.1016/j.autneu.2011.02.004 [doi]

Martina JR et al. (2012) Noninvasive continuous arterial blood pressure monitoring with Nexfin(R) Anesthesiology 116:1092-1103 doi:10.1097/ALN.0b013e31824f94ed

Sato K, Ogoh S, Hirasawa A, Oue A, Sadamoto T (2011) The distribution of blood flow in the carotid and vertebral arteries during dynamic exercise in humans J Physiol 589:2847-2856 doi:10.1113/jphysiol.2010.204461

Sheldon RS et al. (2015) 2015 heart rhythm society expert consensus statement on the diagnosis and treatment of postural tachycardia syndrome, inappropriate sinus tachycardia, and vasovagal syncope Heart Rhythm 12:e41-63 doi:10.1016/j.hrthm.2015.03.029

van Campen CLMC, Verheugt FWA, Rowe PC, Visser FC (2020) Cerebral blood flow is reduced in ME/CFS during head-up tilt testing even in the absence of hypotension or tachycardia: A quantitative, controlled study using Doppler echography Clin Neurophysiol Pract 5:50-58 doi:10.1016/j.cnp.2020.01.003

van Campen CLMC, Verheugt FWA, Visser FC (2018) Cerebral blood flow changes during tilt table testing in healthy volunteers, as assessed by Doppler imaging of the carotid and vertebral arteries Clin Neurophysiol Pract 3:91-95 doi:10.1016/j.cnp.2018.02.004
